# The Association and Mediating Biomarkers of Serum Retinol in Influencing the Development of Type 2 Diabetes: A Prospective Cohort Study in Middle-Aged and Elderly Population

**DOI:** 10.3389/fnut.2022.831950

**Published:** 2022-03-29

**Authors:** Xiuyu Pang, Sen Yang, Xiaoyu Guo, Hongyin Li, Yingfeng Zhang, Chunbo Wei, Yu Wang, Changhao Sun, Ying Li

**Affiliations:** ^1^Department of Nutrition and Food Hygiene, College of Public Health, Harbin Medical University, Harbin, China; ^2^Department of Nutrition and Food Hygiene, School of Public Health, Shandong First Medical University and Shandong Academy of Medical Sciences, Tai’an, China

**Keywords:** type 2 diabetes, nutrition epidemiology, prevention, cohort, serum retinol

## Abstract

The aims of this research are to elucidate whether serum retinol is associated with type 2 diabetes and to explore the underlying mechanisms of the association in a prospective cohort study. A total of 3,526 diabetes-free participants aged 40 years or older were enrolled at baseline in 2010–2012. Multivariable logistic regression was adopted to evaluate the associations of serum retinol and dietary vitamin A (VA) intake with type 2 diabetes. Mediation analyses were used to reveal potential mediators in their associations. After a mean follow-up of 5.3 years, 280 incident cases of type 2 diabetes occurred. Serum retinol was positively associated with the incidence of type 2 diabetes. The multivariable-adjusted odds ratios (ORs) and 95% confidence intervals (CIs) for type 2 diabetes from the bottom to the top quintile of serum retinol were 1, 1.878 (1.202, 2.936), 2.110 (1.364, 3.263), 1.614 (1.027, 2.538), and 2.134 (1.377, 3.306) (*p*-trend = 0.009), respectively. Mediation analysis showed that increased homeostasis model assessment - insulin resistance HOMA-IR, triglycerides (TG), and serum xanthine oxidase (XO) activity could account for 8.5, 14.7, and 12.1% of the total effects of serum retinol on type 2 diabetes, respectively. Serum retinol concentration was not significantly associated with dietary VA intake (*r* = −0.010, *p* = 0.570). In addition, no significant relationship was observed between dietary VA intake and the risk of type 2 diabetes. Overall, elevated serum retinol might increase the risk of type 2 diabetes which is mainly mediated by increased insulin resistance, TG, or serum XO activity.

## Introduction

The dramatically increased prevalence of type 2 diabetes has had a severe impact on morbidity and non-negligible mortality rates, and imposed heavy economic burdens worldwide. Globally, the number of adult patients with diabetes reached 451 million in 2017 and has maintained an incremental trend (1). China is one of the most affected regions, with the prevalence of diabetes reaching 10.9% in 2013, which represents approximately 114 million adults living with diabetes (2).

Given that oxidative stress plays an important role in the development of type 2 diabetes (3), antioxidant vitamins have attracted great interest as they may improve or slow the development of type 2 diabetes (4–7). Unlike vitamin C and vitamin E, the antioxidant properties of vitamin A (VA) have always been controversial (6, 8, 9). Despite the hypothesis that VA may mitigate chronic disease pathogenesis *via* its anti-oxidative properties, several observational studies presented inconsistent findings (8, 10, 11). Mounting evidence suggests that VA probably has pro-oxidant properties in biological systems (12). Thus, the relationship between VA and type 2 diabetes has aroused our interest. Serum retinol concentration is the most commonly used parameter to assess VA status. To date, whether and how serum retinol is associated with type 2 diabetes remain unaddressed issues.

Although several studies reported that the serum retinol levels of patients with diabetes mellitus were within the normal range (13–15), other investigators found that serum retinol was significantly increased in patients with type 2 diabetes compared to control subjects (16). Additionally, serum retinol levels were found to be positively associated with insulin resistance in a prospective cohort of 259 healthy premenopausal women, which indicated that serum retinol might play a role in the pathogenesis of type 2 diabetes (17). Given the contradictory findings from previous studies, it is difficult to determine whether serum retinol is either protective or prodiabetic. Large-scale population cohort studies are urgently needed to explore whether and how retinol is involved in the pathogenesis of type 2 diabetes.

In the present study, we aimed to elucidate whether serum retinol is associated with type 2 diabetes risk by using the long-term follow-up data of the Harbin Cohort Study on Diet, Nutrition, and Chronic Non-communicable Diseases (2010–2015). Furthermore, an improved understanding of the potential mechanisms between serum retinol and type 2 diabetes risk might be beneficial for controlling the development of type 2 diabetes. Thus, we aimed to identify which mediators play critical roles in the total effects of serum retinol on type 2 diabetes risk using mediation analysis after the establishment of certain associations.

## Materials and Methods

### Participants

**FIGURE 1:**
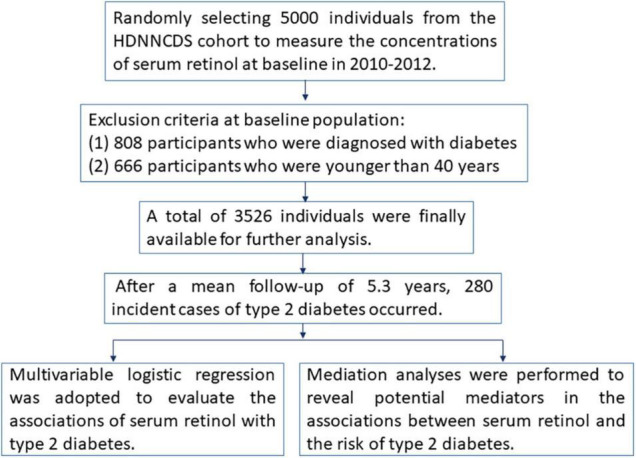
The flowchart of the methodologies in this study.

Subjects in the present study were selected from the Harbin Cohort Study on Diet, Nutrition, and Chronic Non-communicable Diseases (HDNNCDS) prospective cohort study, which has been conducted in Harbin to investigate the impacts of diet and nutrition on chronic non-communicable diseases (18). Figure 1 shows the methodologies of this study in the form of a flow chart. In brief, the cohort study was launched in 2010–2012 and recruited a total of 9,734 people aged 20–75 years (3,526 men and 6,208 women). The first follow-up surveys were conducted in 2015–2016; the average follow-up period was 5.3 years, and the follow-up rate was 91.6%. In this study, we selected 5,000 individuals from the HDNNCDS by employing a completely random design to measure the concentrations of serum retinol at baseline. In our analysis, 808 participants who were diagnosed with diabetes and 666 participants who were younger than 40 years old were excluded. According to the WHO diagnostic criteria (19), type 2 diabetes was diagnosed as FBG ≥ 7.0 mmol/L, and/or 2h-PBG ≥ 11.1 mmol/L, and/or the self-reported use of medication for type 2 diabetes. A total of 3,526 individuals were finally available for further analysis, including 1,178 men (33.41%) and 2,348 (66.59%) women.

### Anthropometric Measurement and Biochemical Assessment

The body mass index (BMI, kg/m^2^) was calculated as body weight (kg) divided by the square of the height (m^2^). Current smokers were defined as those who smoked at least 100 cigarettes in their lifetime and currently smoked every day or on some days. Current drinkers were defined as those who consumed ≥ 1 alcoholic drink each month in the 12 months before the survey (18). Regular exercise was defined as any kind of recreational or sport-related physical activity lasting at least 30 min for more than 3 days per week other than walking for work or in daily life.

Blood samples were collected from all participants who underwent a 75-g oral glucose tolerance test (OGTT). Fasting blood glucose (FBG), 2-h postprandial blood glucose (2h-PBG), total cholesterol (TCHO), triglycerides (TG), low-density lipoprotein cholesterol (LDL-C), and high-density lipoprotein cholesterol (HDL-C) were measured quantitatively with an auto-analyzer (Hitachi 7100 Auto-analyzer, Japan) (20). Fasting insulin (F-insulin) and 2-h postprandial insulin (P-insulin) were measured by immunofluorescence method using an automated enzyme immunoassay (EIA) analyzer (TOSOH AIA-2000ST, Japan) (21). Homeostasis model assessment-insulin resistance (HOMA-IR) was calculated using the following equation: HOMA-IR = (FBG × F-insulin)/22.5 (22).

Serum retinol levels were determined by ultra-high performance liquid chromatography-triple quadrupole mass spectrometry (UPLC-TQ-MS, Waters Corporation, MA, United States) equipped with a C18 column (100 mm × 2.1 mm, 1.7 μm). The standard substance used in the measurement of serum retinol was retinol R7632–100 mg (purchased from Sigma-Aldrich (Shanghai) Trading Co. Ltd., Shanghai, China). The coefficient of variation for the measurement of serum retinol was 7.1%. Given the limitation of blood samples, a total of 1,320 participants without type 2 diabetes at baseline were randomly selected from the HDNNCDS cohort according to age, gender, and BMI to further explore the possible mediation effects of serum xanthine oxidase (XO) activity in the association between serum retinol and type 2 diabetes. Serum XO activity was measured using the Amplex Red reagent method (Molecular Probes, Invitrogen Detection Technologies, Eugene, OR, United States) (23).

### Dietary Assessment

Dietary intake over the past 12 months was assessed *via* the validated food frequency questionnaire (FFQ) at baseline. The validity and reliability of the FFQ were assessed in our previous validation study (18). The dietary VA, lipid, and total energy intakes were estimated by the Chinese Food Composition Tables.

### Statistical Analysis

The distribution of the continuous variables was checked for normality before the analyses. Variables with non-normal distribution (such as serum retinol, HOMA-IR, TG, and serum XO activity) were normalized by a natural logarithm transformation. In all statistical analyses, missing values of covariates were imputed using median values (0.91, 1.7, 6.75, 3.06, 0.57, 0.85, 1.47, and 1.7% of the participants had missing information for being a current smoker, being a current drinker, education level, physical activity, BMI, a history of hypertension, a history of hyperlipidemia, and a history of coronary disease, respectively). Selected demographic characteristics were compared according to type 2 diabetes status using a *t*-test for continuous variables and a chi-square test for categorical variables. ORs and their 95% CIs were estimated to clarify the associations between serum retinol or dietary VA and the incidence of type 2 diabetes based on multivariable logistic regression models. Restricted cubic spline (RCS) regression was applied to detect the possible linear or non-linear dependency of the relationship between the risk of type 2 diabetes and serum retinol levels using 5 knots at prespecified locations according to the 5th, 25th, 50th, 75th, and 95th percentiles of serum retinol. Mediation analysis was performed to examine whether HOMA-IR, blood lipids, and serum XO activity were mediators of the association between serum retinol and the risk of type 2 diabetes. Sensitivity analysis was adopted to test the stability of the mediation analysis. The correlation between serum retinol and dietary VA was analyzed using partial correlation regression analysis.

All statistical analyses were performed using SPSS v 24.0 (IBM Corp. United States) and R version 3.6.1.^1^ Values *p* < 0.05 were considered to indicate statistical significance.

## Results

The demographic characteristics of the participants at baseline in the total sample and by type 2 diabetes status are summarized in Table 1. In the current study, 280 individuals had developed type 2 diabetes during an average follow-up period of 5.3 years. They had higher serum retinol levels than the participants without diabetes. Individuals with type 2 diabetes tended to be older and they had higher BMI, FBG, PBG, F-insulin, P-insulin, HOMA-IR, TG, dietary energy intake, dietary lipid intake, and serum XO activity but lower HDL-C than individuals without diabetes.

**TABLE 1:** Baseline demographic and biochemical characteristics of participants in Harbin Cohort Study on Diet, Nutrition, and Chronic Non-communicable Diseases (HDNNCDS).

Variable	Diabetes (*N* = 280)	Without diabetes (*N* = 3246)	*P*-value
Serum retinol (μmol/L)	1.78 ± 0.92	1.63 ± 0.86	0.005
Age (years)	54.36 ± 7.64	52.44 ± 7.36	<0.001
Male (%)	42.86	32.59	0.001
Current smoker (%)	18.57	14.85	0.099
Current drinker (%)	27.86	31.61	0.202
Exercising regularly (%)	52.86	50.89	0.534
**Education level (%)**			
=High school	78.57	74.37	0.132
>High school	21.43	25.63	
**Physical activity (%)**			
Light	78.57	78.50	0.721
Moderate	18.57	16.57	
Vigorous	1.43	1.73	
Dietary vitamin A intake	776.91 ± 275.37	763.22 ± 262.68	0.405
Dietary energy intake	2492.91 ± 1007.51	2333.83 ± 829.21	0.003
Dietary lipid intake	76.08 ± 27.93	71.02 ± 25.74	0.002
BMI (kg/m^2^)	25.79 ± 3.03	24.89 ± 3.41	<0.001
FBG (mmol/L)	4.95 ± 0.86	4.50 ± 0.67	<0.001
2h-PBG (mmol/L)	7.18 ± 1.96	5.89 ± 1.62	<0.001
F-insulin (μU/mL)	11.58 ± 24.62	8.03 ± 6.89	<0.001
P-insulin (μU/mL)	54.70 ± 43.90	42.64 ± 37.15	<0.001
HOMA-IR	2.60 ± 5.80	1.64 ± 1.51	<0.001
TCHO (mmol/L)	5.20 ± 0.94	5.23 ± 1.01	0.738
TG (mmol/L)	2.17 ± 1.65	1.73 ± 1.51	<0.001
HDL-C (mmol/L)	1.17 ± 0.31	1.28 ± 0.33	<0.001
LDL-C (mmol/L)	3.05 ± 0.87	3.10 ± 0.89	0.338
Serum XO (mU/mL)	3.64 ± 2.65	2.90 ± 2.50	0.001

*Continuous and categorical variables are expressed as the mean ± SD and percentage. The t-test and chi-square test were used to probe for differences in continuous variables and categorical variables. BMI, body mass index; FBG, fasting blood glucose; 2h-BG, 2-h postprandial blood glucose; F-insulin, fasting insulin; P-insulin, 2-h postprandial insulin; HOMA-IR, insulin resistance index, was calculated using the following equation: HOMA-IR = (FBG × F-insulin)/22.5; TCHO, total cholesterol; TG, triglycerides; LDL-C, low density lipoprotein-cholesterol; HDL-C, high density lipoprotein-cholesterol; XO, xanthine oxidase. The standard deviations of F-insulin and HOMA-IR in patients with diabetes were higher than the mean because the data were very discrete. These data were also presented in the form of box-plot and whiskers for clearer presentation of the dispersion information (Supplementary Figure 1).*

**FIGURE 2:**
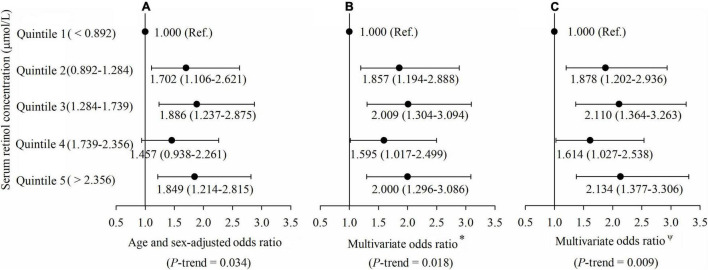
**(A–C)** Adjusted odds ratios (ORs) (and 95% CIs) of type 2 diabetes risk by serum retinol at baseline. *Multivariate ORs were adjusted for age, gender, current smoker, current drinker, exercising regularly, education level, physical activity, dietary vitamin A intake, dietary energy intake, and dietary lipid intake. ^Ψ^Multivariate ORs were adjusted for age, gender, current smoker, current drinker, exercising regularly, education level, physical activity, dietary vitamin A intake, dietary energy intake, dietary lipid intake, body mass index (BMI), hypertension, hyperlipidemia, coronary disease, and a family history of diabetes.

All participants were divided into five groups by quintiles of the concentration of serum retinol, and their risks of type 2 diabetes were compared by multivariable logistic regression in three models. In Model 1 (Figure 2A), the age and sex-adjusted ORs (95% CI) of Quintile 2 (0.892–1.284 μmol/L), Quintile 3 (1.284–1.739 μmol/L), Quintile 4 (1.739–2.356 μmol/L) and Quintile 5 (> 2.356 μmol/L) were 1.702 (1.106, 2.621), 1.886 (1.237, 2.875), 1.457 (0.938, 2.261), and 1.849 (1.214, 2.815), respectively, compared with Quintile 1 (< 0.892 μmol/L), and the *p*-trend was 0.034. In Model 2 (Figure 2B), the multivariate ORs of Quintile 2, Quintile 3, Quintile 4, and Quintile 5 were 1.857 (1.194, 2.888), 2.009 (1.304, 3.094), 1.595 (1.017, 2.499), and 2 (1.296, 3.086), respectively, compared with Quintile 1 after adjusting for age, gender, current smoker, current drinker, exercising regularly, education level, physical activity, dietary VA intake, dietary energy intake, and dietary lipid intake (all at baseline), and the *p*-trend was 0.018. In Model 3 (Figure 2C), the multivariate ORs of Quintile 2, Quintile 3, Quintile 4, and Quintile 5 were 1.878 (1.202, 2.936), 2.110 (1.364, 3.263), 1.614 (1.027, 2.538), and 2.134 (1.377, 3.306), respectively, compared with Quintile 1 after adjusting for BMI, hypertension, hyperlipidemia, coronary disease, and a family history of diabetes, with additional adjustment for the variables mentioned above (all at baseline), and the *p*-trend was 0.009.

To exclude the potential influence caused by prediabetes, a total of 558 prediabetic individuals were excluded based on their FBG or 2h-PBG at baseline. The association of serum retinol with type 2 diabetes was further analyzed in participants with normal blood glucose levels (*N* = 2,968). The findings were consistent with the aforementioned results. As depicted in Supplementary Figure 2, the multivariate ORs of Quintile 2, Quintile 3, Quintile 4, and Quintile 5 were 2.439 (1.264, 4.707), 3.568 (1.907, 6.675), 2.352 (1.225, 4.514), and 3.535 (1.890, 6.613), respectively, compared with Quintile 1 after adjusting for age, gender, current smoker, current drinker, exercising regularly, education level, physical activity, dietary VA intake, dietary energy intake, and dietary lipid intake, BMI, hypertension, hyperlipidemia, coronary disease, and a family history of diabetes, and the *p*-trend was 0.001.

**FIGURE 3:**
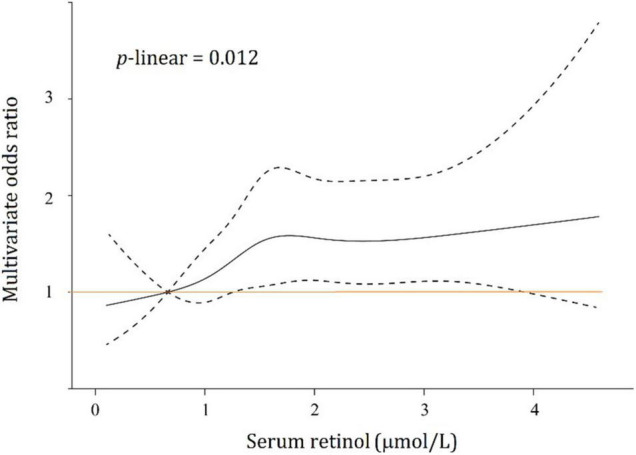
Association between serum retinol and type 2 diabetes risks. Black solid line, multivariate odds ratios; dotted lines, 95% confidence intervals.

The linear or non-linear relationship between serum retinol levels and the risk of type 2 diabetes was modeled by an RCS regression plot with 5 knots after adjusting for all the above potential confounders in multivariable logistic regression (Figure 3). The 5 knots were placed at the 5th, 25th, 50th, 75th, and 95th percentiles of serum retinol which represented 0.477, 0.991, 1.492, 2.156, and 3.296 μmol/L, respectively. Serum retinol was found to be related to diabetes risk in a linear manner (*p*-linearity = 0.012) after adjustment for confounders.

**FIGURE 4:**
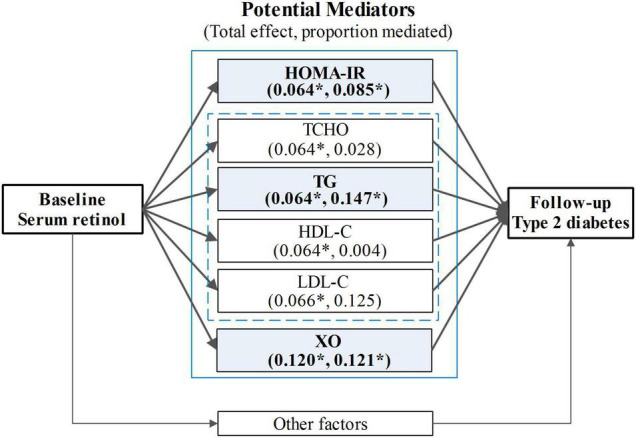
The mediation analysis for the total effect of serum retinol on type 2 diabetes risk. HOMA-IR, the insulin resistance index, was calculated using the following equation: HOMA-IR = (FBG × F-insulin)/22.5; TG, triglyceride; TCHO, total cholesterol; HDL-C, high-density lipoprotein cholesterol; LDL-C, low-density lipoprotein cholesterol; XO, xanthine oxidase. The mediation analysis models were adjusted for age, gender, current smoker, current drinker, exercising regularly, education level, physical activity, dietary vitamin A intake, dietary energy intake, dietary lipid intake, BMI, hypertension, hyperlipidemia, coronary disease, and a family history of diabetes. *p < 0.05.

A mediation analysis was employed to examine the mediation effects and the proportion of mediating effects (including HOMA-IR, TCHO, TG, TC, HDL-C, HDL-C, and serum XO activity) in the association between serum retinol levels and type 2 diabetes at follow-up (Figure 4). In this study, three of the biomarkers (HOMA-IR, TG, and serum XO activity) were found to mediate statistically significant proportions in the total effects. A sensitivity analysis was performed to assess the robustness of the mediating effects, and the results showed that the sensitivity for all three of the aforementioned mediators in the mediation analysis was relatively stable (Table 2). Insulin resistance (indicated by HOMA-IR), which served as a necessary cause of type 2 diabetes, mediated 8.5% of the total effects. As an indicator of serum lipids, TG is a direct risk factor for type 2 diabetes, and it mediated 14.7% of the total effects. Serum XO activity, an independent risk factor for type 2 diabetes, significantly mediated the serum retinol–type 2 diabetes relationship (accounting for 12.1% of the total effects of serum retinol on type 2 diabetes risk). However, the mediation effects of TCHO, HDL-C, and LDL-C were insignificant. In these mediation analysis models, all of the aforementioned potential confounders in the multivariable logistic regression were also adjusted.

**TABLE 2:** The sensitivity analysis for mediation analysis.

Mediators	The parameters of sensitivity analysis	
	R2*	R∼2
HOMA-IR	0.010	0.007
TG	0.010	0.008
Serum XO activity	0.010	0.008

HOMA-IR, insulin resistance index, calculated using the following equation: HOMA-IR = (FBG × F-insulin)/22.5; TG, triglycerides; serum XO, serum xanthine oxidase. R2* and R∼2 are two parameters indicating the quality of sensitivity analysis.

**FIGURE 5:**
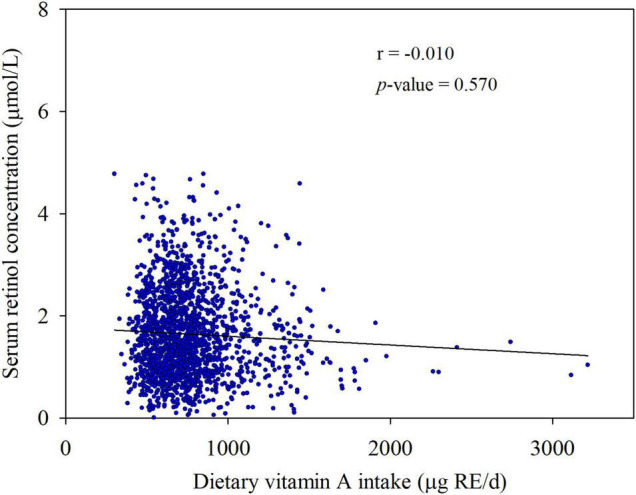
Associations between serum retinol concentration and dietary vitamin A intake. Partial r and *p*-values were obtained after adjustment for age, gender, current smoker, current drinker, exercising regularly, education level, physical activity, dietary energy intake, dietary lipid intake, and BMI.

**FIGURE 6:**
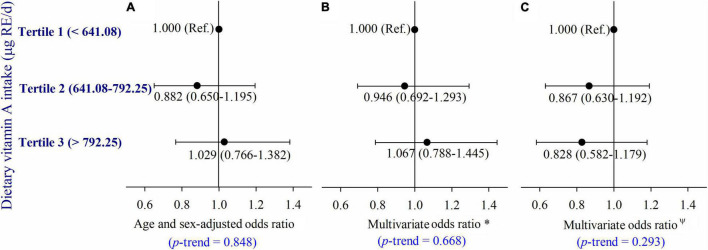
**(A–C)** Adjusted ORs (and 95% CIs) of type 2 diabetes by dietary vitamin A intake at baseline. *Multivariate ORs were adjusted for age, sex, current smoker, current drinker, exercised regularly, education level, physical activity, BMI, hypertension, hyperlipidemia, coronary disease, and a family history of diabetes; ^Ψ^Multivariate ORs were adjusted for age, gender, current smoker, current drinker, exercising regularly, education level, physical activity, BMI, hypertension, hyperlipidemia, coronary disease and a family history of diabetes, dietary energy intake, and dietary lipid intake.

Serum retinol was not significantly associated with dietary VA intake in the partial correlation analyses (*r* = −0.010, *p* = 0.570) after adjusting for age, gender, current smoker, current drinker, exercising regularly, education level, physical activity, dietary energy intake, dietary lipid intake, and BMI (Figure 5). Moreover, we did not observe a significant relationship between dietary VA intake and the risk of type 2 diabetes (Figure 6). The participants were divided into three groups by tertiles of dietary VA intake, and their ORs for type 2 diabetes were calculated by multivariable logistic regression in three models. In Model 1 (Figure 6A), the age- and gender-adjusted ORs (95% CI) of Tertile 2 and Tertile 3 were 0.882 (0.650, 1.195) and 1.029 (0.766, 1.382), respectively, compared with Tertile 1 (*p*-trend = 0.848). In Model 2 (Figure 6B), the multivariate ORs of Tertile 2 and Tertile 3 were 0.946 (0.692, 1.293) and 1.067 (0.788, 1.445), respectively, compared with Tertile 1 after adjusting for age, gender, current smoker, current drinker, exercising regularly, education level, physical activity, BMI, hypertension, hyperlipidemia, coronary disease, and a family history of diabetes (*p*-trend = 0.668). The ORs remained insignificant after additional adjustments for dietary energy intake and dietary lipid intake in Model 3 (Figure 6C). The multivariate ORs of Tertile 2 and Tertile 3 were 0.867 (0.630, 1.192) and 0.828 (0.582, 1.179), respectively, compared with Tertile 1 (*p*-trend = 0.293).

## Discussion

In this large-scale prospective study, we observed that serum retinol levels were positively associated with the incidence of type 2 diabetes in middle-aged and elderly Chinese adults. In addition, increased insulin resistance, TG, and serum XO activity mediated the association. To the best of our knowledge, the current study is the first to reveal the underlying mechanisms of the association between serum retinol and type 2 diabetes risk in a cohort study.

In this study, a positive association of serum retinol with the incidence of type 2 diabetes was observed, which was mostly consistent with a number of small studies of humans (24, 25). However, there have also been human studies reporting contradictory findings of the relationship between serum retinol and type 2 diabetes (26, 27). The contradictory findings from these studies, coupled with the limitations of small-scale and cross-sectional human studies, made it difficult to determine whether serum retinol was either protective or prodiabetic. Indeed, our findings, which are based on a large-scale prospective cohort study, add an important piece of evidence.

How serum retinol contributes to the pathogenesis of type 2 diabetes is an unresolved issue. In this study, insulin resistance index (28) (indicated by HOMA-IR), serum lipids (including TCHO, TG, HDL-C, and LDL-C) (29), and serum XO activity (30) were included in the mediation analysis because all of them might be potential mediators that play vital roles in the pathogenesis and development of type 2 diabetes.

Serum retinol presented a positive association with insulin resistance (being a necessary cause of type 2 diabetes). The mediation analysis revealed that insulin resistance could mediate the effect of serum retinol on the risk of type 2 diabetes. Similar results were observed by Blondin et al., who found that serum retinol was positively associated with insulin resistance and was considered an important factor in the pathogenesis of type 2 diabetes in a small-scale prospective cohort study (17). In addition, higher serum retinol concentrations were also found in subjects with impaired glucose tolerance and showed a positive correlation with insulin resistance in a previous population study (31). Corbetta et al. (32) observed a mild but significant elevation of insulin resistance after retinoid therapy. All the above findings support our results that increased insulin resistance may play a critical mediating role in the total effects of serum retinol on the risk of type 2 diabetes. Moreover, we found that the effect of serum retinol on the risk of type 2 diabetes could also be mediated by serum TG. Serum retinol appeared to be positively related to TG in the present population study, which was consistent with previous animal and human studies in which serum retinol was found to be correlated with serum lipid levels (33). There was no doubt that higher TG levels could increase the risk of type 2 diabetes. Retinoids can elevate serum TG levels by increasing Apo C-III expression (34), which serves as an antagonist of serum TG catabolism. In addition, hypertriglyceridemia appears to be a predisposing factor for hypervitaminosis A (10), while VA has the disadvantage of causing hepatotoxicity and an elevation in serum TG (35). Farhangi et al. conducted a randomized controlled trial of VA supplementation in women of reproductive age who were obese. They found that the participants who received VA alone or in combination with β-carotene supplementation had significantly higher TG levels than those in the placebo group (36). The aforementioned results indicated a possible role of serum TG in mediating the effect of serum retinol on type 2 diabetes risk. Apart from insulin resistance and serum TG, we found that serum XO activity could also mediate the effect of serum retinol on type 2 diabetes risk. XO, apart from its role in uric acid production, can generate oxidants, and thus promote the pathogenesis of diabetes (31). In our previous cohort study, we observed that serum XO activity was an independent risk factor for type 2 diabetes (22). In addition, retinol was found to promote reactive species production by elevating XO activity in plenty of *in vitro* studies (12, 37), which was consistent with our hypothesis that increased oxidative stress caused by elevated XO might be an important link between retinol and type 2 diabetes risk. Of course, all of the aforementioned findings warrant further attention and need further validation in cell, animal, and human studies.

In fact, although serum retinol was found to be positively related to the incidence of type 2 diabetes in middle-aged and elderly Chinese adults, this does not mean that we recommend reducing the intake of VA in the daily diet. We found that the serum retinol levels were not directly related to dietary VA intake, which was consistent with previous studies (38, 39), but that they mainly depended on nutritional habits, lifestyle, and the body’s metabolic state. Moreover, as we observed, the intake of dietary VA presented an insignificant protective influence on type 2 diabetes risk after multivariable adjustment. This was basically consistent with the findings of Eshak et al., who reported that dietary VA intake tended to be associated with a reduced risk of type 2 diabetes in an age- and gender-adjusted model in a Japanese population, but the trend went to null after multivariable adjustment (8). In another study carried out in Finland, an increased risk of diabetes was observed in male smokers with high dietary VA consumption; however, the association disappeared after multivariable adjustment (40). Consistent with several previous studies, this study indicated that there were no significant associations between serum retinol and dietary VA (38, 41). Of course, there are also studies reporting high serum retinol levels in patients with extremely high VA intake, in which 49 and 73% of the patients exceeded the current recommendations (38). Woestenenk et al. suggested that the correlation with serum retinol levels is minimal or non-existent with moderate VA intake, while with a very high intake, serum retinol levels might indeed be boosted to above normal levels (41). This supposition was also indicated in our study. Thus, we proposed that serum retinol and dietary VA should be researched and discussed separately instead of together. In this study, although we found that elevated serum retinol can increase the risk of type 2 diabetes, this does not mean that we recommend reducing the intake of dietary VA.

Thus, the relation of serum retinol level to type 2 diabetes risk and that of dietary VA intake to type 2 diabetes risk should be treated in a different view. Our research population was mainly composed of older adults, and these individuals were susceptible to chronic diseases, such as type 2 diabetes. We speculated that a high serum retinol level was an external presentation of high-level oxidative stress in the body, which was probably induced by the internal metabolic hazard. In addition, serum retinol seems appropriate to serve as an important indicator to reflect the status of the metabolic state or oxidative stress in the current study. Metabolic disorder and high-level oxidative stress, which could increase the risk of type 2 diabetes, are increasingly prevalent, and their incidences are increasing rapidly around the world (2). Accordingly, we propose that chronic diseases, especially type 2 diabetes, should be taken into account when reconstructing the criteria for assessing VA status.

The current large-scale prospective cohort study investigating the underlying mechanisms of the association between serum retinol and type 2 diabetes provides credible references for further research owing to its small bias. However, this research also has certain limitations. As the research was carried out based on an Asian population, the extension of our findings needs further verification. In addition, considering the limitations of epidemiological studies, a range of animal and *in vitro* experiments are required to validate the potential mechanisms in the associations.

## Conclusion

In conclusion, elevated serum retinol showed a positive correlation with the incidence risk of type 2 diabetes in middle-aged and elderly Chinese populations. Insulin resistance, TG, and serum XO activity might mediate the effect of serum retinol on type 2 diabetes. Our research provides a better understanding of the role of serum retinol in the development of type 2 diabetes from a new perspective, which is believed to advance the management of type 2 diabetes. We suggest that the criteria for recommending serum VA status should not only consider deficiency diseases; risk brought by elevated serum retinol to chronic diseases, especially type 2 diabetes, should also be emphasized for the wisdom of public health.

## Data Availability Statement

The original contributions presented in the study are included in the article/Supplementary Material, further inquiries can be directed to the corresponding author/s.

## Ethics Statement

The study was approved by the Ethics Committee of the Harbin Medical University and had been conducted in accordance with the Declaration of Helsinki. Written informed consent was provided by all participants. This study was registered at chictr.org as ChiCTR-ECH-1200272119. The patients/participants provided their written informed consent to participate in this study.

## Author Contributions

XP designed the study, performed the data analyses, elaborated the tables and figures, and drafted the manuscript. SY, XG, and HL contributed to the statistical analyses and manuscript revisions. YZ and CW carried-out the main examination. CS and YW contributed to the revision of the manuscript. YL was the guarantor of this study, supervised the study, and revised the manuscript. All authors gave their final approval.

## Conflict of Interest

The authors declare that the research was conducted in the absence of any commercial or financial relationships that could be construed as a potential conflict of interest.

## Publisher’s Note

All claims expressed in this article are solely those of the authors and do not necessarily represent those of their affiliated organizations, or those of the publisher, the editors and the reviewers. Any product that may be evaluated in this article, or claim that may be made by its manufacturer, is not guaranteed or endorsed by the publisher.

## Footnotes

1
http://www.r-project.org/
