# Supplementary material for: The Association and Mediating Biomarkers of Serum Retinol in Influencing the Development of Type 2 Diabetes: A Prospective Cohort Study in Middle-Aged and Elderly Population
Source: Front Nutr. 2022 Mar 29;9:831950. doi: 10.3389/fnut.2022.831950 (PMC9002104; doi:10.3389/fnut.2022.831950)
Supplement: Supplementary file 1 [file Data_Sheet_1.docx]

**SUPPLEMENTARY MATERIALS**

**The association and mediating biomarkers of serum retinol in influencing the development of type 2 diabetes: A prospective cohort study in middle-aged and elderly population**





**Figure S1. The Box-plot of F-insulin and HOMA-IR at baseline.**





**Figure S2. Adjusted odds ratios (ORs) (and 95% confidence intervals) of type 2 diabetes risk by serum retinol at baseline.**

Multivariate ORs were adjusted for age, gender, current smoker, current drinker, exercising regularly, education level, physical activity, dietary VA intake, dietary energy intake and dietary lipid intake, BMI, hypertension, hyperlipidemia, coronary disease and a family history of diabetes.
